# Reuse of patients’ own drugs in hospitals in Ghana; the evidence to support policy

**DOI:** 10.1186/s12913-018-3860-9

**Published:** 2019-01-11

**Authors:** Pauline Boachie-Ansah, Berko Panyin Anto, Afia Frimpomaa Asare Marfo

**Affiliations:** Department of Pharmacy Practice, Faculty of Pharmacy, KNUST, Kumasi, Ghana

**Keywords:** Patient own drugs, Pharmacists, Hospital pharmacists, Policy, Teaching hospital

## Abstract

**Background:**

Given the documented benefits of Patient Own Drugs (PODs) in most developed countries and scanty data on PODs management in developing countries the aim of the study was to evaluate the assessment, quality and extent of PODs use among hospitalised patients. Furthermore the perceived benefits and challenges in executing PODs management by the pharmacy staff in the hospital setting were explored.

**Method:**

This was a cross-sectional descriptive study. Three hundred patients with chronic diseases admitted in a teaching hospital were purposively sampled. Quality assessment criteria was developed as part of the data collection tool for assessing the quality of PODs. Furthermore, two ward pharmacists and two in-charge nurses at the medical ward were purposively sampled for a face to face interview using an interview guide to find out the hospitals’ medicines management system and policy for PODs. In addition, 130 pharmacy staff were interviewed using a structured questionnaire to find out how PODs were managed. Data was analysed with SPSS version 17.

**Results:**

The study showed that 140 (46.6%) of patients brought their PODs on admission. Of these, only 38 (12.7%) were told to bring them whenever they were on admission. Of the 115 (38.3) patients whose PODs were documented as part of medication history, 28 (24.3%) of them had their PODs continued whilst on admission and 11(9.5%) of discharged prescription included PODs. In assessing the quality of PODs 61.6% of 845 PODs were suitable for reuse. Only 19.8% of pharmacy staff attested to the fact that all PODs identified were assessed. The common benefit of PODs cited by pharmacy staff was improving medication history taking whilst the major challenge was difficulty in determining the expiry dates of PODs without original packages.

**Conclusion:**

About a half of patients with chronic diseases brought PODs with them on admission. The majority of PODs appeared to be suitable for use as presented but only a few were actually used for the patients. Most pharmacy staff were not involved in patients own drugs management at the hospital. There is the need for a policy to streamline PODs management in the teaching hospital.

**Electronic supplementary material:**

The online version of this article (10.1186/s12913-018-3860-9) contains supplementary material, which is available to authorized users.

## Background

Patients’ own drugs (PODs) are medicines that patients have obtained in community setting or through a prescription at hospital and brought to hospital when admitted [1]. For patients with chronic diseases PODs may be continued whiles on admission [2]. Normally PODs may be used in the hospital, when the quality had been approved by a laid-down assessment criteria by the pharmacist, the prescriber had written an order for that same medicine and the hospital policy permits the use of PODs [3]. In most hospitals in the United Kingdom where PODs schemes have been initiated, pharmacy ward based technicians and pharmacists are responsible for assessing the quality of PODs and streamlining the patients’ discharge list especially with their PODs [4].

In health systems where PODs schemes have been implemented several benefits have been documented. These include lessened wastage of medicines, improved accuracy of admission orders, reduced medication-related adverse events and lowered treatment costs with better treatment outcomes. The scheme has also reduced length of stay and readmission rates, opportunities for patient counselling and continuity of care [5]. A research conducted at the Evelina Children’s Hospital, London found that of the patients who brought their PODs to the hospital, 80% used them during their stay with no change in dosage. In addition 84% were discharged with one or more PODs whilst 50% were discharged on the same POD [6]. It has been suggested that the use of PODs is a potential avenue for reducing medication errors. However, one review measuring medication administration errors made by nurses found out that there were no significant differences in the errors made when PODs were used compared with the traditional system of using only hospital pharmacy-dispensed prescriptions [7].

In Ghana, medicines are estimated to constitute 60–80% of the cost of health care [8]. As a result inappropriate use and management of medicines may have medical and social implications and exert undue financial burden on the health care system as well as on patients and the country at large. Furthermore, policies regarding the use of PODs are non-existent, not to mention its management in hospitals. Therefore, this study seeks to provide evidence that reuse of PODs on hospital admission is an ongoing process to inform policy to support its effective management. The current role and perceived challenges of PODs management by the pharmacy staff would be explored. In addition the potential for pharmacists’ involvement in terms of opportunities for efficient medicine management would also be explored.

## Method

### Study design and setting

This was a cross-sectional descriptive study. Both quantitative and qualitative methods were adopted for the study. The narrative approach was used for the qualitative aspect of the study. This approach was used because we wanted to have an in-depth knowledge on the availability and operations of a PODs policy at the hospital. The study was conducted in four medical wards at the Komfo Anokye Teaching Hospital (KATH), a tertiary care institution in Kumasi, the Ashanti region of Ghana. This setting was chosen because the Komfo Anokye Teaching Hospital is a referral hospital that serves the northern sector of the country.

### Study population

For the quantitative aspect of the study the inclusion criteria were in-patients aged 18 years and above, with a known chronic medical illness (es) and being managed with medications. Severely ill, unconscious and mentally ill patients were excluded. The sample size of three hundred patients was chosen assuming that 50% of the in-patients come along with their PODs to the hospital in the past year, and then using a margin of error 0.05 and a confidence level of 95%. In addition all pharmacy staff (pharmacists, pharmacy technicians, and one dispensary assistant and pharmacy interns) working in the pharmacy units of the hospital were systematically sampled and interviewed. Two clinical pharmacists and two in-charge nurses at the medical ward were purposively sampled for the qualitative study.

### Development and validation of research instrument

Two research instruments were developed for the collection of quantitative data (Additional file 1). The first instrument was a two-sectioned questionnaire was developed by the researchers to collect the appropriate medical data from patients who were included in the study. The first section was designed to collect bio-data of the patients including their gender, age, level of education, occupation, past medical, drug history, diagnosis, current list of medicines on the ward and discharge medicines. Section two basically focused on the availability, quantity and quality of PODs. The assessment for quality of PODs was based on the evaluation of the appearance of PODs, storage container, labelling, and expiration of PODs (Table 1). The Quality checklist for assessment of PODs was adapted from the study by Fradgley and Pryce (2002) and PODs assessment criteria by NHS Tayside, UK [7, 8].

**Table 1:** Quality checklist criteria for Patients’ Own Drugs (PODs)

Label	
• It must present, legible and clean	
• It must state the name, the strength and actual quantity of drug	
• Instructions should be clear and understandable	
• Medicines should have been dispensed within the time frame its remain efficacious.	
Container	
• The container should be original, intact and clean	
• There should only be one product within the container	
Expiry	
• The expiry date should be found and should not be exceeded.	
Product Integrity	
• The medicine should be easily identified.(especially if clear liquid or loose white tablets)	
• The product should be whole, clean with no visible deterioration (eg, no broken tablet)	
Storage	
• The medicine must be stored according to manufacturer’s recommendations	

Adapted from: Quality checklist for assessment of PODs by Fradgley and Pryce (2002) and PODs assessment criteria by NHS, UK

The questionnaire was also face validated by two academic pharmacists and a clinical pharmacist. Furthermore it was piloted and weaknesses amended accordingly.

The second instrument was a structured questionnaire, designed for the collection of data from the pharmacy staff. This questionnaire consisted of questions that sought to find out whether PODs was managed with a structured guideline, factors considered when assessing PODs, perceived benefits and challenges in the management of PODs and suggestive ways to improve it. The questionnaire for the pharmacy staff was piloted at a continuous education programme for pharmacists and pharmacy technicians in Kumasi-Ghana. This helped with the clarity in the definition of PODs and restructuring of the questionnaire.

For the qualitative study two clinical pharmacists and two in-charge nurses at the medical ward purposively sampled were face to face interviewed using an interview guide. Topics covered included sources of PODs, assessment of PODs, disposal of unused PODs and whether the hospital had a written policy on PODs management.

### Data collection & analysis

Data was collected from 1st to the 31st of May, 2014. Patient recruitment was done on daily basis. All patients on admission were eligible for recruitment based on our inclusion criteria. Patients gave their consent before they were recruited into the study. On the first day of admission information on demographic and clinical characteristics and medication history was collected from the folder and interaction with the patient or carer. After that PODs available were assessed based on the set criteria. The PODs of each patient was supposed to pass all the five components of the hospital. Patients who had PODs at home were asked to bring it and these were assessed. Patients on admission were followed up till discharge so as to have a record of medicines the patients were discharged on.

All pharmacy staff involved in the study were also administered their questionnaire to be filled on their own and the questionnaires were collected after a week.

The structured questionnaire for the patients and pharmacy staff were coded, stored and analysed using the Statistical Package for Service Solutions (SPSS) version 20.0 and Microsoft Office Excel 2010. The Fishers exact test was used to compare the availability of PODs at the hospital with the sex and duration of the patient’s illness. A *p* value less than 0.05 was defined as statistically significant.

For the Qualitative Study the interview was audio recorded, transcribed and grouped into themes. The audio recording from the interviews was transcribed and analysed using a qualitative data analysis software N-vivo version and Microsoft excel. Transcription was validated by comparing transcripts to notes taken during the interview.

## Results

### Demographic and clinical characteristics of inpatients

Three hundred out of 493 in-patients were purposively sampled during the study period. One hundred and forty (46.7%) were males. A high proportion of participants were aged 18-35 years (*n* = 83, 27.6%) and 51-65 years (*n* = 84, 28.1%). One hundred and two participants (34.0%) had no education whilst 101(33.6%) had basic education. Two Hundred and seventy (90%) of the participants were enrolled on the National Health Insurance Scheme (NHIS) of Ghana. Chronic illness presented by participants included hypertension (*n* = 32, 10.7%), diabetes (*n* = 28, 9.3%), renal disorders (*n* = 11, 3.7%), liver diseases (*n* = 3, 4.3%). The average inpatient hospital stay was 6.5 ± 3.2.

### Availability of PODS

One hundred and fifty two participants (50.6%) brought PODs to the hospital, 109(36.3%) left PODs at home and 39 (13.1%) had PODs finished before they came on admission. Of the 152 participants who brought PODs to the hospital 43(28.2%) were asked to take them home and 23(15.1%) had PODs in their bags without the awareness of the hospital staff. Furthermore only 38 participants who brought their PODs to the hospital were told to bring it along any time they came on admission. Reason cited by participants for bringing PODs to the hospital although they were not told include therapy change 17(14.9%), to save money 25(21.9%) and continuity 62 (54.4%). Participants left PODs at home because of forgetfulness 4 (45.0%) and fear of bringing PODs 60(55%) to the hospital as they were not told to bring their medicines to the hospital. Fischer’s exact test run showed a statistically significant association between the availability of PODs at the hospital and patient’s duration of illness. [OR, 5.143 95% CI, 3.998–14.586, *p* = 0.003]. Patients who have had the condition for more than five years were likely to bring their medicines to the hospital. However, there was no association between the availability of PODs at the hospital and the gender of patients. [OR, 2.133 95% CI, 2.678–10.454, *p* = 0.076].

### Assessment of the quantity, quality and extent of use of PODs

All available PODs brought by participants and those left at home but brought by relatives upon request were assessed by the researcher. A total of 845 PODs were identified. A high proportion of participants had two and three PODs (Table 2). None of the PODs brought to the hospital by the participants for reuse was re-labelled by any of the hospital staff.

**Table 2:** Quantity of PODs per Participant

Number of PODs per in-patient	Number of participants, N (%)
1	55 (18.3)
2	71 (23.7)
3	56 (18.7)
4	23 (7.7)
5	18 (6)
More than 5 PODs	47 (15.7)
Finished before admission	30 (10)
Total	300 (100)

Of the 152 participants who brought their PODs, it was observed that 24% of them had their PODs continued on the first day of admission with no supply of medicines from the pharmacy. Although 53% of participants were prescribed medicines which included PODs on the first day new medicines were supplied. Reasons accounting for this were that patients had PODs in their bags without the awareness of the hospital staff and the physician was not comfortable with the use of PODs. Twenty three percent of patients had their PODs discontinued as there was no similarities between PODs and medicines prescribed on the first day of admission. Nine percent of patients had PODs continued during discharge, with no supply from the pharmacy. Although PODs were included in 55.7% of patient’s prescriptions new medicines were supplied from the pharmacy. There were no similarities between PODs and medicines prescribed during discharged among 33.3% of patients.

One hundred and thirty six of the participants had PODs which passed the quality assessment criteria. In all 486 (57.5%) out of 845 PODs assessed passed the quality assessment test. Table 3 illustrates the results of the assessment of five components of the assessment criteria. Reasons why some PODs did not pass quality assessment were that PODs were not in the original blister pack, not labelled and faded instructions on plastic envelopes containing loose tablets.

**Table 3:** The Assessments of Quality of the PODs

Components		Number PODS	
		PODs available at the hospital N (%)	PODs made available at the hospital based on request from the researcher N (%)
Label present on the container of PODs	Yes, complete	254 (30.1)	155 (18.3)
	Yes, incomplete	203 (24)	125 (14.8)
	No	67 (7.9)	41 (4.9)
Instruction for use	Legible	247 (29.2)	151 (17.9)
	Illegible	277 (32.9)	170 (20.1)
PODs clean and intact	Yes	500 (57.2)	307 (36.3)
	No	24 (2.8)	14 (4.9)
Expiry date identified	Yes	467 (39.5)	286 (33.8)
	No	57 (6.7)	35 (4.1)
PODs expired	Yes	38 (4.5)	24 (2.8)
	No	453 (53.6)	277 (32.9)
	Not Sure	33 (3.9)	20 (2.4)

The total cost savings to the hospital with the use of PODs over the period of the study was US$100. However the total cost of 486 PODs which passed the quality assessment test and suitable for reuse was US$1673.

## Pharmacy staff survey

Responses were received from 121 pharmacy staff out of 130 who were administered the questionnaire. These included 57 pharmacists, 47 pharmacy technicians, one dispensary assistant and 16 pharmacy interns. Sixty-seven were males and 54 females. Thirty eight (31.0%) pharmacy staff had seen patients who brought their PODs to the hospital. Furthermore 41 pharmacy staff (33.9%) were of the view that the hospital had no policy/guidelines for the management of PODs of in-patients while 70(57.9%) could not tell if such a policy existed. None of the 10 (8.3%) pharmacy staff who said the hospital had a policy could prove it with a written policy or guideline. Furthermore 49 pharmacy staff (40.5%) said no assessments of PODs were made before reuse and 48 (39.7%) could not tell if PODs were assessed at the hospital. None of the 24(19.8%) pharmacy staff who confirmed that PODs identified were assessed by a pharmacy staff before any decisions were made, could tell how or who exactly was in-charge of assessing PODs.

However perceived factors to be considered when PODs are assessed cited by pharmacy staff were physical quality, expiry dates, legibility of instructions on the label and storage conditions of PODs. Improvement in medication history taking [89(67%)] and creation of an avenue for medication counselling were some benefits of PODs cited by respondents. Sixty two (51%) and 44 (36%) of pharmacy staff strongly agreed that patients having different brands of the same medicine and illegible instructions on medicines label could be a challenge to PODs management respectively. (Table 4).

**Table 4:** Perceived Challenges in the Management of PODs

Variables	Strongly agree N (%)	Agree N (%)	Uncertain N (%)	Disagree N (%)	Strongly disagree N (%)	MEDIAN (IQR)
Patient having different brands of the same medicines	62 (51)	37 (31)	4 (3)	17 (14)	–	1 (1.00)
Inadequate time for PODs assessment at the ward	21 (17)	57 (47)	19 (16)	19 (16)	5	2 (1.00)
Lack of POD Policy in the hospital	28 (23)	54 (45)	33 (27)	6 (5)	–	2 (1.00)
Inadequate skills for staff for the assessment of PODs in in-patients	10 (8)	45 (37)	10 (8)	32 (26)	24 (20)	3 (2.00)
It must be a shared responsibility amongst the pharmacy staff	41 (34)	58 (48)	12 (10)	10 (8)	–	2 (1.00)
Patients feel they should always be given new medicines	19 (16)	50 (41)	21 (17)	30 (25)	–	2 (2.00)
Illegible instructions on label	44 (36)	50 (41)	20 (17)	7 (6)	–	2 (1.00)
Difficulty in examining the expiry dates of PODs without original packages.	44 (36)	57 (47)	8 (7)	12 (10)	**–**	2 (1.00)
Inadequate staff to assess PODs of all in-patients	40 (33)	36 (30)	20 (17)	25 (21)	–	2 (2.00)
The illiteracy level of in-patients/carers	43 (36)	32 (26)	25 (21)	17 (14)	4	2 (2.00)
Inadequate education/ counselling to patients to bring their PODs when coming to the hospital	45 (37)	53 (44)	11 (9)	12 (10)	–	2 (1.00)

## Hospitals PODs Management system and policy

These results were obtained from the qualitative study.

Sources of PODs mentioned by the clinical pharmacists and ward nurses when interviewed were from home and other hospitals.

*When patients are transferred from other hospitals they bring their medicines along* **WN 1.**

*Some patients with chronic illness also bring their medicines from home when they are on admission* **CP 2**

From the perspective of the ward nurses and clinical pharmacists interviewed, the decision on the continuation or cessation of PODs is normally done by the doctor on duty and this is documented in the patient’s folder. Further there is no written procedure for the assessment and storage of PODs, disposal of unused PODS and there is no policy guiding the management of PODs in the hospital.

*‘The prescriber often states in the patient’s folder that the patient has the medicine and it should be continued. When PODs are stopped relatives are asked to take them home or it is kept in either the patients’ medicine box or in the left over box on the ward’.* ***WN1***

*‘There is no laid down criteria for the assessment of PODs. When the prescriber states in the patients’ folder that PODs should be given the medicines are served with no assessment’.* ***WN2***

*‘The pharmacy department does not often manage patients own drugs available on the ward. The pharmacist does not access the quality of these medicines and also the pharmacy department does not dispose of unused PODs. Medicines prescribed on the ward are often submitted to the pharmacy to be dispensed’.* ***CP1***

*‘There is no guideline for the management of patients own drugs at the hospital. Patients bring their medicines to the hospital at their own discretion. Patients admitted on the ward are rarely asked to bring their medicines from home’.* ***CP2***

*WN: WARD NURSE CP: CLINICAL PHARMACIST.

## Discussion

### Availability of PODS

About 50% of participants had one or more PODs available during admission. Although this is encouraging all patients on long term medicines admitted to the hospital should be encouraged to bring their own medicines as this could aid in the medication history documentation and possibly reduce medication errors. However some participants who brought PODs were asked to take them home because therapy was not continued and some also had it in their bags without the knowledge of the hospital staff. In similar studies discontinued PODs were not returned to the patients because study sites had in place guidelines on the disposal of unused PODs [9]. In this study an appreciable number of participants left their PODs at home because they did not know that they had to bring their medicines to the hospital. In most institutions where patient own medicines management scheme are run, the scheme is often advertised and patients are made aware [10, 11].

### Assessment of quantity, quality and extent of use of PODs

A total of 845 PODs were counted when all PODs including those at home were collected. According to the Ghana National Drug Programme (GNDP), the minimum information to appear on the label shall consist of the: name of the patient, generic name of the drugs, strength of the active ingredient, quantity of dispensed product, complete dose regimen in written and/or graphic form, name and address of the dispensing facility and dispenser special instructions, date of dispensing and duration of use [12]. About 57% of PODs passed the quality assessment test and suitable for reuse. This compares well with a study in the United Kingdom where 60% of PODs were suitable for reuse [13]. However about 40% of in-patients with PODs did not pass the assessment test, because no re-labelling was done during admission. Some in-patients had PODs which did not pass the assessment criteria test although they were continued on admission indicating the need for a guideline on the assessment of PODs. Out of the 24% participants who had their PODs continued on admission, 9% had PODs as part of their discharge medicines. This similarity between PODs on the first day of admission and discharge prescriptions further reaffirms the need for PODs management in our hospitals so that their suitability for re-use could be assessed. Furthermore, since the PODs were continued on admission the hospital made some savings on medicines and also wastage was reduced. However these benefits witnessed could have increased if a PODs scheme was operational at the hospital.

### Pharmacy staff survey

Pharmacists have the responsibility to ensure the safe, effective and rational use of medicines and as such play a vital role in the delivery of health care worldwide. This study showed that pharmacy staff were marginally involved in the assessment of PODs although they were aware of most of the factors that should be considered when PODs are assessed for reuse. It’s very important that pharmacy staff are encouraged to be involved in patients own drug management because if patients’ medicines are checked on admission by the ward pharmacist or technician or any healthcare staff who is properly trained, their suitability for reissue could be assessed and waste could be prevented.

The participation of all stakeholders from healthcare personals through to patients in medicine management processes can help to generate a rational for the monitoring of medicine with maximal cost benefits. Most pharmacy staff were aware of the benefits of PODs which is very encouraging as this could enhance their involvement in PODs management if the right polices are put in place.

More than 50% (an average of all the agreements) of the pharmacy staff agreed that they were more likely to encounter the out-listed possible challenges in various degrees when they had to assess and manage PODs. It could be seen from their response that the issue of inadequate skills to assess PODs were not that much of a challenge to the staff probably because the educational syllabus of the pharmacy school had addressed them. However some practical issues such as additional workload for the pharmacy staffs, defining roles and responsibilities, effective communication with health providers and the public, the need for criteria to assess PODs and staff training should be addressed if PODs management schemes are to be formalised.

### Hospital PODs management system and policy

Currently there is no hospital policy on PODs management from the perspective of ward pharmacists, nurses and pharmacy staff. There is the need for the management of the teaching hospital to draft a policy on PODs to streamline the management of patient own drugs brought to the hospital as PODs management scheme implemented in most of the developed countries have shown positive outcomes [14, 15]. Outcomes such as cost effectiveness of the schemes would be very beneficial in our developing settings where healthcare cost financing is always an issue. It is also very essential that the PODs policy provides details on advertisement of policy to patient, assessment of PODs brought (when, how and by whom) to the hospital and the management of PODs that are no longer needed by the patient. This is because in the study managing unused PODs brought from the house by relatives was an issue as there is no policy. However discussions were made and the pharmacy manager of the pharmacy unit asked that the PODs should be collected and brought to the pharmacy, if the patient or his careers agreed.

There are potential avenues for the active involvement of pharmacy staff in the PODs management. Pharmacy staff could be involved in the effective education and counselling for patients/relatives on the need to bring their PODs to hospital and assess the quality of PODs on the ward for it suitability for reuse. With regard to the assessment of the quality of PODs, the criteria used in our study could be adopted. In addition health institutions in developing countries who are interested implementing PODs management schemes could also adopt our criteria for assessment.

### Strength and limitation of the study

The strength of the study is its novelty in Africa; it is the only study in Africa that have assessed the management of PODs in a health institution. The results of this research cannot be generalised to all hospitals in Ghana because the study was conducted in a teaching hospital. However the results obtained could be similar to other teaching hospitals. The limitation of the study was that it was a single site study. Furthermore the fact that the questionnaires were left with the pharmacists for one week before collection was also a limitation. Some challenges were also encountered during the assessments of the PODs. Some PODs were outside the original blister pack, not labelled and faded instructions on plastic envelopes containing loose tablets. Other challenges were inaccurate medication list, no laid down policy for the disposal of unwanted PODs and improper ward storage of PODs.

## Conclusions

Majority of PODs were of good quality but only few were actually used in the hospital because the hospital does not have a policy for the management of patient owns drugs. Furthermore just about half patients brought their own medicines to the hospital because most patients were not aware of any PODs scheme at the hospital and also assessment of PODs was not often done hence patient kept PODs in their bags without the knowledge of the staff. However the hospital made some savings on the use of PODs and wastage was reduced as some PODs were continued on the first day of admission and during discharge. In addition the hospital would have made more savings if a PODs policy was operational. Although the pharmacy staff are aware of the benefits of PODs they are not involved in the management of patient own drugs.

A written guideline and policy on PODs and an effective education and counselling for patients on the need to bring their PODs could help improve the management of PODs at the hospital and the advantage of using PODs could also be manifested in the hospital.

## Additional file

Additional file 1: Data collection form. (PDF 305 kb)

## Abbreviations

CHRPE: Committee on Human Research Publication and Ethics
CP: Clinical Pharmacist
KATH: Komfo Anokye Teaching Hospital
KNUST: Kwame Nkrumah University of Science and Technology
PODs: Patient Own Drugs
R&D: Research and Development Unit
SMS: School of Medical Sciences
WN: Ward Nurse
