# Supplementary material for: Reuse of patients’ own drugs in hospitals in Ghana; the evidence to support policy
Source: BMC Health Serv Res. 2019 Jan 11;19:27. doi: 10.1186/s12913-018-3860-9 (PMC6329137; doi:10.1186/s12913-018-3860-9)
Supplement: Supplementary file 1 — Data collection form. (PDF 305 kb) [file 12913_2018_3860_MOESM1_ESM.pdf]

# DATA COLLECTION FORM FOR PHARMACY STAFF

**KWAME NKRUMAH UNIVERSITY OF SCIENCE AND TECHNOLOGY (KNUST)**

**DEPARTMENT OF CLINICAL AND SOCIAL PHARMACY**

**EVALUATING THE MANAGEMENT OF PATIENTS' OWN DRUGS (PODs) AT THE MEDICAL WARDS OF KOMFO ANOKYE TEACHING HOSPITAL (KATH) – KUMASI**

Kindly tick [√ ] the response that best corresponds to your opinion.

1. Gender Male [ ] Female [ ]
2. Please indicate your level of qualification

   Pharmacist [ ] Pharmacy Technician [ ] Dispensary assistant [ ] Pharmacy Intern pharmacist [ ] Other [ ], specify……………………………………..
3. How many years have you practiced?

   Less than 5yrs[ ] 5-10yrs [ ] Rarely [ ] No [ ] Don't know [ ]
4. Do patients bring along their medicines when coming to the hospital?

   Yes [ ] No [ ]
5. Is it important for patients bring their medicines when coming to the hospital?

   Yes [ ] No [ ]
6. Which of the following do you see as benefits of the management of POMs?

| **Possible benefits of the management of PODs** | **Strongly agree** | **Agree** | **Uncertain** | **Disagree** | **Strongly disagree** |
|---|---|---|---|---|---|
| It serves as strategy to prevent medication error | | | | | |
| To reduce the complexity of patients' medicines | | | | | |
| Improving accuracy in medication history taking | | | | | |
| Creates avenue for education and counseling | | | | | |
| It helps to identify if admission was due to prescribing errors or adverse reactions to medicines bought from the community | | | | | |
| Helps to lower treatment costs | | | | | |

7. Does the hospital have any written policy about the handling of PODs of patients on admission?

   Yes [ ] No [ ]
8. Are there any quality assessment check made on the available PODs?

   Yes [ ] No [ ]

9. Which of the following factors would you consider when assessing the POMs of each in-patient? Please tick [ √ ] as appropriate.

| **Factors to consider when assessing PODs of in-patients** | **Yes** | **No** | **Not Sure** |
|---|---|---|---|
| The physical integrity of the PODs | | | |
| The quantity of the PODs | | | |
| The expiry dates on the PODs | | | |
| The storage conditions patient gives to PODs | | | |
| Seeking the consent of the patients before disposing an unsuitable POD | | | |
| Re-labelling and documentation of PODs that are continued on admission | | | |
| Checking whether PODs are part of Discharge medicines | | | |
| Providing education for the patient/carer upon admission, about the hospital's take on the use of PODs at the ward | | | |

10. For each of the statement below, tick [ ] the response which best corresponds to the your thinking on the possible challenges that might be encountered in the management of PODs at your facility.

| **Possible challenges to the management of POMs** | **Strongly agree** | **Agree** | **Uncertain** | **Disagree** | **Strongly disagree** |
|---|---|---|---|---|---|
| Difference brands of same medicines procured and dispensed | | | | | |
| Inadequate time for PODs assessment at the ward | | | | | |
| Lack of PODs policy guidelines in the hospital | | | | | |
| Inadequate skills for staff for the assessment of PODs | | | | | |
| It is a shared responsibility of the pharmacy staff | | | | | |
| Patients on NHIS feel they should always new medicines | | | | | |
| The illegibility of the labels on medicines | | | | | |
| Inadequate staff to assess PODs of all in-patients | | | | | |
| The illiteracy level of patients/carers | | | | | |
| Inadequate counseling/education given to patients/carers to bring along all their PODs when coming to the hospital | | | | | |

11. What in your opinion could be done to improve the management of PODs in patients on admission at this hospital? ……………………………………………………………………

# DATA COLLECTION FORM FOR IN-PATIENTS

**KWAME NKRUMAH UNIVERSITY OF SCIENCE AND TECHNOLOGY (KNUST)**

**DEPARTMENT OF CLINICAL AND SOCIAL PHARMACY**

**EVALUATING THE MANAGEMENT OF PATIENTS' OWN DRUGS (PODs) AT THE MEDICAL WARDS OF KOMFO ANOKYE TEACHING HOSPITAL (KATH) – KUMASI**

Patient's code: ………… Ward: …………… Bed No……………………..

Admission Date: ……… Diagnosis………… Discharge Date: ……………..

Data Collected by: ............................................... Date collected: ………………

1. Gender Male[ ] Female [ ]
2. Age……………………………………….
3. Level of education ……………………….
4. Occupation
5. Are you on the NHIS? Yes [ ] No [ ]
6. What sickness do you have probably caused your admission to this ward? .....................................
7. Kindly provide the following information about your own medicines (whether prescribed or supplementary) that you were taking before coming on admission.

| Name & strength of PODs | PODs available[√] or unavailable [x] | Your best description of your PODs | Are PODs captured in the DHx in their folder (Y/N) |
|---|---|---|---|
| | | | |
| | | | |
| | | | |

Kindly give (where possible) the **reasons** to the statements below.

8. PODs available on admission………………………………………………………
9. PODs are not available on admission……………………………………………….
10. The use of other supplementary medicines (if only patient has)…………………………
11. Would you like to be given your POD if it were prescribed for you again either on admission or as part of your discharge medicines? Yes [ ] No [ ]

## APPENDIX V: ASSESSMENT FORM FOR PODS

| ASSESSMENT CRITERIA FOR PATIENTS' OWN DRUGS AVAILABLE | | | | |
|---|---|---|---|---|
| Name & strength of POD(s) | | | | |
| | Yes, complete | | | |

| | | | | |
|---|---|---|---|---|
| Label present | Yes, incomplete | | | |
| | No | | | |
| Instructions for use legible | Legible | | | |
| | Illegible | | | |
| Clean and intact | Yes | | | |
| | No | | | |
| Expired date identified? | Yes | | | |
| | No | | | |
| POD expired? | Yes | | | |
| | No | | | |
| | Not Sure | | | |
| Quantity left | | | | |
| PODs part of first day Rx | | | | |
| PODs part of discharged Rx | | | | |

## QUALITY CHECKLIST CRITERIA FOR PATIENTS' OWN DRUGS (PODs)

### Label

- It must present, legible and clean
- It must state the name, the strength and actual quantity of drug
- Instructions should be clear and understandable
- Medicines should have been dispensed within the time frame its remain efficacious.

### Container

- The container should be original, intact and clean
- There should only be one product within the container

### Expiry

- The expiry date should be found and should not be exceeded.

### Product Integrity

- The medicine should be easily identified.(especially if clear liquid or loose white tablets)
- The product should be whole, clean with no visible deterioration (eg, no broken tablet)

### Storage

- The medicine must be stored according to manufacturer's recommendations

**Adapted from:** Quality checklist for assessment of POD (Panel 1) by Sean et al and PODs assessment criteria by NHS Tayside Medicines Management I (Use of POD)
